# The Effects of Appropriate Perioperative Exercise on Perioperative Neurocognitive Disorders: a Narrative Review

**DOI:** 10.1007/s12035-023-03864-0

**Published:** 2023-12-19

**Authors:** Hao Feng, Zheng Zhang, Wenyuan Lyu, Xiangyi Kong, Jianjun Li, Haipeng Zhou, Penghui Wei

**Affiliations:** Department of Anesthesiology, Qilu Hospital (Qingdao), Cheeloo College of Medicine, Shandong University, Qingdao, People’s Republic of China

**Keywords:** Exercise, Perioperative neurocognitive disorders, Postoperative delirium, Postoperative cognitive dysfunction, Mitochondria, Neuroinflammation, Gut microbiota

## Abstract

Perioperative neurocognitive disorders (PNDs) are now considered the most common neurological complication in older adult patients undergoing surgical procedures. A significant increase exists in the incidence of post-operative disability and mortality in patients with PNDs. However, no specific treatment is still available for PNDs. Recent studies have shown that exercise may improve cognitive dysfunction-related disorders, including PNDs. Neuroinflammation is a key mechanism underlying exercise-induced neuroprotection in PNDs; others include the regulation of gut microbiota and mitochondrial and synaptic function. Maintaining optimal skeletal muscle mass through preoperative exercise is important to prevent the occurrence of PNDs. This review summarizes current clinical and preclinical evidence and proposes potential molecular mechanisms by which perioperative exercise improves PNDs, providing a new direction for exploring exercise-mediated neuroprotective effects on PNDs. In addition, it intends to provide new strategies for the prevention and treatment of PNDs.

## Introduction

Perioperative neurocognitive disorders (PNDs) are associated with cognitive changes after anesthesia and surgery, particularly in older adults. They are defined as alterations in neuropsychiatric function that occur in the perioperative period, including preoperative cognitive impairment, postoperative delirium (POD), delayed neurocognitive recovery (dNCR), and postoperative cognitive dysfunction (POCD) [1]. PNDs can significantly prolong patient hospitalization and increase post-operative patient disability and mortality and aggravate the burden of patient and family medical care, leading to serious social problems [2].

In the latest recommendations, PNDs are classified into several different stages based on specific time intervals [1]. Specifically, POD refers to neuropsychiatric disorder characterized by inattention, disorganized thinking, and altered levels of consciousness, which is associated with cognitive impairment and commonly diagnosed in the first week following surgery. The term “dNCR” refers to cognitive dysfunction within 30 days after the procedure. POCD is defined as neurocognitive disorders during the period from 30 days postoperatively to 12 months. The incidence of POD is between 5.1 and 30.3% in patients who undergo different procedures [3–5]. Multiple studies have shown that the incidence of dNCR after major non-cardiac surgery is approximately 25–34.5%. However, most of the patients with early post-operative cognitive decline recover over time, and only one-fifth to one-third develop POCD during follow-up [6, 7]. Unfortunately, no existing drugs can effectively treat this disorder because its mechanism remains unclear. Animal and human studies have proposed several hypotheses about the pathophysiology of PNDs and have developed novel treatments based on these hypotheses [8]. However, currently, available treatment options are limited and appear unable to reduce the mortality and incidence rate of PNDs. Furthermore, studies have shown that physical activity prevents and reduces cognitive impairment and improves cognitive function [9]. Therefore, non-pharmacological treatment options, such as exercise, are promising alternative intervention strategies for preventing and treating PNDs in older adults. This works through the inhibition of neuroinflammation, regulation of gut microbiota, maintenance of skeletal muscle mass, amelioration of mitochondrial dysfunction, and modulation of synaptic plasticity. This review summarizes recent progress in exercise and PNDs to provide a theoretical basis and potential molecular mechanism for preventing and treating PNDs and evidence for developing relevant clinical trials.

## Exercise Decrease the Risk and Severity of POD

Preoperative exercise capacity as an independent predictor of POD has been demonstrated in elective cardiac surgery. Ogawa et al. [10] found that the 6-minute walking distance (6MWD) test results can be used for predicting incidence. The cut-off value of 6MWD determined by the receiver operating characteristic (ROC) curve was 345 m. Therefore, POD prevention may be achieved by improving the outcome of 6MWD during preoperative rehabilitation exercises. In another retrospective study, preoperative low physical activity was suggested in patients with gastrointestinal cancer as a predictor of POD, independent of confounding factors [11]. Similarly, preoperative low skeletal muscle mass (LSMM) [12] and exercise intensity [13] are associated with POD in older adult patients undergoing colorectal cancer (CRC) surgery. Patients with LSMM can be identified based on gender and skeletal muscle index (SMI) by assessing the cross-sectional area of skeletal muscle at the third lumbar vertebra (L3) level on computed tomography (CT) [14]. LSMM was associated with POD based on multivariate analysis, and a significantly stronger association was found in patients who were malnourished or physically dependent and undergoing CRC surgery. Additionally, patients with sarcopenia have a higher risk of developing delirium after a major thoracolumbar spine surgery [15]. A retrospective study explored the relationship between skeletal muscle mass and POD in patients undergoing surgery for head and neck cancer, and a decrease in SMI was found to be a significant independent risk factor for hypoactive and mixed-type POD in these patients [16]. Preventing such POD may be possible in free flap repair for oral cancer by increasing skeletal muscle mass preoperatively through exercise and nutritional therapy. Lee et al. showed that in patients undergoing elective orthopedic surgery, regular preoperative exercise significantly reduced the risk of POD and was associated with the severity of POD in a secondary analysis of prospective studies. Interestingly, the effects of exercise on POD appeared to have significant gender differences [17]. According to the above studies (Table 1), the skeletal muscle mass and preoperative exercise capacity can be improved by proper preoperative exercise to prevent POD in elderly patients. Furthermore, determining the preoperative exercise capacity to predict and prevent POD is an essential novel research direction.

**Table 1:** The effects of exercise on postoperative delirium: human studies

Study	Participants	Age, mean (SD), or median (IQR), year	Study design	Exercise-related indicator	Surgical procedures	Assessment tool	Results
Ogawa et al. [10]	313 patients	68.6 (± 14.8)	Single-center, prospective cohort study	6MWD; TUG	Elective cardiac surgery	ICDSC	6MWD was a statistically significant indicator for POD (OR 0.98; *p* = 0.02).
Yanagisawa et al. [11]	178 patients	70 (IQR, 63–77)	Retrospective study	Physical activity	Primary gastrointestinal cancer surgery	Short CAM	The association between preoperative physical activity and POD are significant (OR 2.83; *p* = 0.03).
Mosk et al. [12]	251 patients	76 (IQR, 73–80)	Retrospective observational cohort study	SMI	Colorectal cancer surgery	DOSS	POD occurred significantly more in patients with LSMM (25%) compared with patients without LSMM (10%), *p* = 0.006.
Xu et al. [13]	122 patients	72.77 ± 5.38	Retrospective study	Exercise intensity	Ileostomy for colorectal cancer	MoCA and FACT-Cog	Exercise intensity is independent predictors of postoperative cognitive impairment.
Makiguchi et al. [16]	122 patients	60.3 (± 11.2)	Nonrandomized, retrospective cohort study	SMI	Free flap repair after oral cancer resection	DSM-IV	Lower SMI was identified as significant independent risk factors for POD (OR 2.52; *p* = 0.035).
Lee et al. [17]	132 patients	70.5 (IQR, 64.5–76.3)	Prospective, single-center, cohort study	Frequency and type of exercise	Elective orthopedic surgery	CAM	Regular exercise (6–7 days per week) was associated with 74% lower odds of POD (odds ratio [OR] = 0.26; 95% CI = 0.08–0.82)

*POD* postoperative delirium; *6MWD* 6-minute walking distance; *TUG* timed up-and-go test; *ICDSC* intensive care delirium screening checklist; *SMI* skeletal muscle index; *DOSS* delirium observational screening scale; *LSMM* low skeletal muscle mass; *DSM-IV* diagnostic and statistical manual of mental disorders, 4th edition; *CAM* confusion assessment method; *MoCA* Montreal cognitive assessment; *FACT-Cog* functional assessment of cancer therapy-cognitive function; *CAM* confusion assessment method; *OR* odds ratio

## Mechanisms of Exercise Interventions for POD and POCD

The common mechanism of cognitive dysfunction is irreversible neuronal loss and dysfunction. Currently, the known factors contributing to PNDs include neuroinflammation, mitochondrial dysfunction, and reduction of brain-derived neurotrophic factors (BDNF). In preclinical studies, exercise has a significant positive impact on PNDs by regulating neuroinflammation, gut microbiota, skeletal muscle, mitochondria, and synapses (Table 2).

**Table 2:** The effects of exercise on perioperative neurocognitive disorders: preclinical studies

Study	Model system	Exercise modality	Duration	Frequency	Evaluated criteria	Significant outcomes
Feng et al. [18]	LCR or HCR rat	Treadmill	6 weeks	31.5 min/day 5 days/week	TFC; MWM; hippocampal IL-6, HMGB-1, MCP-1, Itgax, Netrin-1; diversity of gut microbiota	▴Spatial learning, memory on MWM. Freezing behavior on TFC ▾Hippocampal IL-6, HMGB-1, MCP-1, Itgax, Netrin-1 ▴Diversity of gut microbiota
Lai et al. [19]	C57BL/6 J mouse	Treadmill	4 weeks	30–40 min/day 5 days/week	BMT; NOR; gut microbiota profiling; SCFA; hippocampal IL-1β, IL-6, GDNF, IBA-1, PSD95, C3ar1, brain cell genesis, dendritic; diversity of gut microbiota	▴Spatial learning, memory on BMT and NOR ▴Diversity of gut microbiota ▾Hippocampal IL-6, IBA-1, C3ar1, PSD95, GDNF ▴Hippocampal dendritic branches ▾Valeric acid
Liu et al. [20]	C57BL6/N mouse	Resistance training	4 weeks	15 times/day	NOR; Y-maze test; hippocampal MCP-1, IBA-1, GFAP, PGC1-α, BDNF, mitofusin-2, mitochondria density, percentage of abnormal mitochondria, NMDA receptor, dendritic arborization and spine density	▴Spatial learning, memory on Y-maze test, and NOR ▾Hippocampal MCP-1, IBA-1, GFAP, percentage of abnormal mitochondria, mitofusin-2, NMDA receptor, and AMPK activation ▴Hippocampal PGC1-α, BDNF, mitochondria density, dendritic arborization and spine density.
Nemoto et al. [21]	Sprague-Dawley rat	Tail suspension (non-exercise)	2 weeks	Every day	MWM; fear conditioning test; hippocampal neurogenesis and BDNF	▾Spatial learning, memory on MWM. Freezing time on fear conditioning test. Hippocampal neurogenesis and BDNF

*GDNF* glial cell-derived neurotrophic factor, *PSD95* postsynaptic density protein 95, *C3ar1* complement 3a receptor 1, *BMT* Barnes maze test, *NOR* novel object recognition, *IBA-1* ionized calcium binding adaptor molecule 1, *SCFA* short chain fatty acids, *LCR* low capacity runner, *HCR* high capacity runner, *TFC* trace fear conditioning, *MWM* Morris water maze, *HMGB-1* high mobility group protein 1, *MCP-1* monocyte chemoattractant protein-1, *BDNF* brain-derived neurotrophic factors, *GFAP* glial fibrillary acidic protein, *NMDA N*-methyl-D-aspartic acid, *AMPK* adenosine 5′-monophosphate (AMP)-activated protein kinase

### Neuroinflammation

The body’s innate immune system, as the primary defense system, is vital for fighting infections and exogenous pathogens. Nevertheless, in the absence of infection, the innate immune system may be triggered by endogenous danger signals, such as damage-associated molecular patterns (DAMPs) [22]. When the immune system identifies an exogenous or endogenous trigger, immune cells recognize and eliminate them through cytokine release, antigen presentation, and phagocytosis [23, 24]. DAMPs can be activated to act on pattern recognition receptors (PRRs) to activate the intrinsic immune system after aseptic mechanical injuries such as a surgical operation, which in turn causes a multi-protein signal transduction cascade response and secretion of pro-inflammatory factors. Activated macrophages can penetrate across the damaged blood–brain barrier (BBB) and further produce inflammatory cytokines in the hippocampus, resulting in neuroinflammatory responses and neuronal cell injury [25]. An over-activated immune system may lead to chronic neuroinflammation, thus adversely impacting the central nervous system (CNS).

As immune cells in the CNS, microglia can differentiate into two types after being activated: M1 phenotype and M2 phenotype. “M1 microglia” mainly produce pro-inflammatory cytokines to exert neurotoxic effects, such as interleukin-1β (IL-1β). Conversely, “M2 microglia” are critically involved in the anti-inflammatory response and promote tissue homeostasis and extracellular matrix reconstruction [26]. Some neurodegenerative diseases, however, cause chronic neuroinflammatory conditions by adversely activating glial cells due to persistent pathological formations [27, 28]. Owing to the “non-specific” innate immune system, such chronic inflammation and overactivation of glial cells have been classified as detrimental processes. It has been shown that these responses cause damage to other surrounding neurons as well as glial cells, delaying the recovery of disease [29].

After peripheral surgery, multiple humoral and neural pathways modulate the interactions between the immune response and the brain. Various cytokines secreted from the periphery enter the CNS across the disrupted BBB to activate microglia [30]. Furthermore, in a prospective cohort study of patients undergoing non-intracranial surgery, it was observed that a peripheral inflammatory response that increased with the degree of surgical trauma was able to exacerbate breakdown of the BBB and thereby induce POD [31]. In addition, in another clinical study, patients diagnosed with POD after undergoing elective spine surgery had higher levels of pro-inflammatory cytokines compared to non-POD patients [32]. In a preclinical study, not only was the inflammatory response exacerbated in the periphery and hippocampus, but behavioral tests performed poorly with increasing time the mice underwent surgery [33]. Surgical stress can alter the dynamic balance of microglia activation by shifting to the M1 phenotype, creating a vicious cycle of neuroinflammatory progression and inducing consequent cognitive dysfunction [34, 35]. Importantly, modulation of neuroinflammation not only attenuates POD-like behavior induced by anesthesia and surgery in elderly mice [36] but also prevents the development of POD in older adult patients undergoing non-cardiac surgery [37].

Exercise has been found to have benefits for neurodegenerative diseases and cognitive function by influencing markers of inflammation systemically, including the CNS. It is noteworthy that the impact on peripheral inflammatory factors varies depending on the type of exercise. In clinical study, endurance exercise reduces levels of proinflammation in the periphery, while resistance and concurrent exercise only decrease TNF-α levels, which may be linked to a reduction in adiposity [38]. In preclinical studies, short- and long-term free wheel running exercise decreased IL-6 levels in the CNS in aged mice [39]. Short-term resistance training inhibits the activation of microglia and astrocytes in the hippocampus in Alzheimer’s disease (AD) mice. It also reduced pro-inflammatory cytokine levels and increase the level of anti-inflammatory mediators in the brain, thereby improving cognitive function in AD mice [40].

Treadmill exercise induces microglial M1-to-M2 polarization shift in APP/PS1 mice, which in turn inhibits pro-inflammatory response and promotes anti-inflammatory response, improving cognitive dysfunction in mice through suppression of the neuroinflammatory response [41]. In a separate study, compared with high-capacity runner (HCR) rats, low-capacity runner (LCR) rats undergoing isoflurane anesthesia and experiencing tibia fracture with internal fixation exhibited excessive cognitive impairment at 3 days and at 3 months postoperatively, which could be improved by preoperative treadmill exercise lasting 6 weeks. Furthermore, treadmill training in LCR rats significantly attenuates the increase in protein and mRNA expression of pro-inflammatory cytokines in post-operative LCR rats [18]. Similarly, an additional preclinical study showed that anesthesia and surgery increased microglia activation [42] and pro-inflammatory cytokines in the hippocampus, and exercise attenuated this increase [19]. These results reveal that exercise can modulate neuroinflammatory responses on CNS and can reduce the risk of suffering from PNDs.

### Gut Microbiota

Gut microbes are microorganisms that exist in the gastrointestinal system in a symbiotic relationship [43]. The gut microbiota communicates with the brain in a bidirectional manner through multiple pathways, thereby establishing the gut–brain axis, which has recently been shown to play a critical role in many neurological disorders [44]. In a microbial community, the α diversity index represents species richness and the relative abundance. The β diversity index estimates the diversity of species in different environments and indicates species’ response to the environment. Several taxa have been revealed to be involved in altered cognitive function in the gut microbiota. The genus *Streptococcus* serves an essential role in causing neurological damage and is also a risk factor for cognitive decline [45]. As an essential member of the gut microbiota, Lachnospiraceae may improve cognitive impairment and inflammatory responses in the hippocampus by producing metabolites [46]. Moreover, decreased abundance of Trichophytonaceae in the gut microbiota is correlated with a higher frequency of cognitive impairment [47].

Alterations in gut microbiota composition are involved in mice’s anesthesia and surgery-induced cognitive impairment. Liu et al. observed that aging mice exhibited more significant POD-like behavior after undergoing anesthesia and surgery compared to young mice. This appears to be related to a more significant reduction in the relative abundance of *Lactobacilli* in the gut microbiota of elderly mice. By feeding probiotic not only improved the inflammatory response in the hippocampus but also improved cognitive function after anesthesia and surgery in aging mice [48]. In sevoflurane-treated young mice, Lachnospiraceae exhibited an increase in relative abundance, whereas Trichophytonaceae exhibited a decrease in relative abundance [49]. In a prospective clinical study, it was concluded by analyzing the gut microbiota of elderly patients after surgery, in which *Parabacteroides distasonis* was associated with POD. However, *Prevotella* and *Colinella* were not statistically associated with the occurrence of POD [50]. This appears to imply that alterations of gut microbiota diversity are influenced by factors such as age, species, and mode of intervention. Therefore, identifying PNDs may be possible through quantitative analysis of specific intestinal bacteria and will facilitate the development of new therapeutic strategies based on these findings.

Exercise can induce neurochemical changes in the brain by modulating the gut microbiota. Voluntary exercise ameliorates high-fat diet-induced cognitive dysfunction by elevating the relative abundance of *Lactobacillus*, and *Eubacterium nodatum* in the intestinal flora of mice [51]. Similarly, in APP/PS1 mice, persistent treadmill exercise for 12 weeks increased the diversity of the intestinal flora and also increased the relative abundance of probiotics thereby declining AD-associated pathological markers [52]. Furthermore, in a randomized controlled trial, 12 weeks of moderate-intensity training modulated the abnormal composition of gut microbiota and improved depressive symptoms [53]. Therefore, regulating the gut microbiota through exercise is a promising therapeutic direction to ameliorate diseases caused by abnormal disorders of gut bacteria.

Exercise can ameliorate the adverse impact of anesthesia and surgery on cognitive function through the modulation of gut flora metabolites. Short-chain fatty acids (SCFAs) are products of the gut microbiota [54]. The concentration of valeric acid, a SCFA, increased in the blood of mice undergoing anesthesia and surgery, and preoperative exercise can attenuate this increase [19]. Brain structural modifications, such as neurogenesis and synaptic structure, are often necessary for learning and memory [55]. The intraperitoneal administration of valeric acid reversed exercise’s beneficial impact on mice’s cognitive performance after anesthesia and surgery. Moreover, the decrease in pro-inflammatory cytokines, increase in brain cell genesis, and improvement in synaptic plasticity after anesthesia and surgery caused by exercise were also attenuated by intraperitoneal injection of valeric acid [19]. In general, these findings reveal that valeric acid, derived from gut microbiota, significantly promoted PND progression, which could be addressed through exercise.

### Skeletal Muscle Mass

Sarcopenia is a degenerative change in skeletal muscle mass and strength with age, resulting in reduced muscle physiology [56]. Approximately 10–40% of people over the age of 60 years suffer from sarcopenia [57]. Sarcopenia may lead to adverse clinical outcomes, decreased self-care ability, and mortality [58]. The current literature indicates a strong correlation between sarcopenia and neurodegeneration [59], such as AD [60] and brain atrophy [61]. Moreover, POD is associated with muscular atrophy caused by sarcopenia [12].

A kind of neurotrophic factor, BDNF, promotes neurogenesis and regulates synaptic plasticity [62]. Specifically, BDNF performs multiple functions by binding to its tropomyosin receptor kinase B (TrkB); these functions include supporting neuronal survival and growth, affecting synaptic transmission, enhancing neurogenesis, and altering synaptic plasticity [63]. Skeletal muscle has been found to be a secretory organ that is a major source of BDNF [59]. Furthermore, exercise increases the level of BDNF according to previous studies [64], and BDNF functions with exercise to promote hippocampal neurogenesis and improve cognitive decline in mice [65]. Treatment with TrkB inhibitors blocked exercise’s beneficial effects in Parkinson’s disease (PD) rats [66]. However, it is unclear how muscle-derived BDNF crosses the BBB to affect the brain. FNDC5 is a muscle protein induced by exercise that can be cleaved and converted into iridin [67]; interestingly, the expression of BDNF increases when irisin is upregulated in primary cortical neurons, whereas it is reduced when FNDC5 is knocked down with RNAi [68]. Furthermore, hippocampal BDNF and other neuroprotective genes were increased due to the elevated expression of FNDC5 and irisin in peripheral blood [68]. In addition, exercise induces an increase in myokine and cathepsin B, which can be released into the peripheral blood and cross the BBB to increase protein and mRNA expression of BNDF in the hippocampus [69].

Resistance exercise prevents the decline in BDNF and improves POCD in aged mice undergoing anesthesia and surgery, indicating that BDNF is important in regulating the beneficial effects of exercise on POCD [20]. Nemoto et al. reported a low skeletal muscle mass animal model by suspending the rats’ tail for 2 weeks without allowing the hind limbs to touch the ground [21]. The analysis excluded rats who tried climbing their own tails due to insufficient muscle atrophy. In this experiment, the MWM and fear conditioning test results demonstrated that rats with low skeletal muscle mass undergoing anesthesia and surgery showed cognitive decline. Additionally, inhibition of the proliferation of immature nerves in the hippocampal dentate gyrus and a reduction in BDNF were also revealed. Thus, maintaining optimal muscle strength and mass through preoperative exercises is important to prevent the occurrence of PNDs.

### Mitochondria

Mitochondria increase in number through biogenesis and fusion processes, thereby increasing their capacity for energy supply and Ca^2+^ handling [70]. However, fission and mitophagy occur when mitochondria are excessive or dysfunctional to maintain cellular metabolic homeostasis and mitochondrial health [71]. Mitofusin-1/2 (Mfn1/2) and optical atrophy protein 1/2 (Opa1/2) are core proteins in the mitochondrial fusion mechanism; they can promote the fusion of the outer and inner mitochondrial membranes, respectively [72]. Proteins, dynamin-related protein 1 (Drp1), and fission protein 1 (Fis1) combine to complete mitochondrial fission, ultimately leading to contraction and cleavage of mitochondrial segments for clearance [73]. Mitophagy, which is more efficient when fission occurs [74], mediates the removal of damaged mitochondria through autophagy and lysosomal degradation, thus ensuring intracellular mitochondrial quality.

Increased release of reactive oxygen species (ROS) and cell death–related factors caused by impaired mitophagy mechanism play an important role in neurodegenerative diseases [75]. Increased ROS resulting from mitochondrial dysfunction can over-activate microglia to cause neuroinflammation and apoptosis [76]. Cognitive dysfunction induced by chronic cerebral underperfusion in rats is mainly attributed to increased ROS and neuroinflammation due to mitochondrial dysfunction, and this pathological process was reversed by stabilizing mitochondrial function and promoting mitochondrial biogenesis [77]. Anesthesia and surgery promote ROS accumulation and inhibit energy metabolism in the brain of aging mice, primarily by disrupting the balance of mitochondrial dynamics and altering mitochondrial ultrastructure [78]. Similarly, the activation of mitochondrial fission caused by anesthesia and surgery resulted in increased mitochondrial fragmentation in the hippocampus of aged mice [42].

Several studies have shown that exercise may increase the number of mitochondria and improve oxidative phosphorylation and respiratory capacity [79, 80]. Mice receiving swim training showed improved cognitive function, inhibited oxidative stress, and increased expression of Mfn1/2 and Drp1 compared to untrained mice [81]. In another study, 12 weeks of treadmill exercise increased mitochondrial fusion proteins and mitochondrial autophagy proteins and inhibited fission-related proteins, resulting in improved mitochondrial respiratory activity and reduced apoptotic signaling in the cerebral cortex and cerebellum [82]. Transplantation of plasma from long-term exercised mice into AD mice improves spatial memory and learning performance by improving mitochondrial function and inhibiting apoptosis in the brain, which appeared to correlate with high levels of BDNF in the plasma of exercised mice [83]. In summary, all the above results indicate that exercise can maintain brain health by improving mitochondrial dynamics.

Four weeks of preoperative resistance exercise in mice improved cognitive dysfunction induced by laparotomy [20]. Moreover, preoperative resistance exercise reversed the hippocampal mitochondrial defects induced by laparotomy in aged mice. Furthermore, anesthesia and surgery can increase Mfn-2 levels in the hippocampus, which can be prevented by resistance exercise, suggesting that resistance exercise also ameliorates post-operative cognitive impairment in aged mice by moderating mitochondrial dynamics [20].

### Synapse

The synapse is where neurons transmit information to each other, which is primarily mediated by neurotransmitters [84]. Dendritic spines protrude from the dendritic shaft, the main site of synaptic plasticity [85]. Synaptic plasticity involves the formation of new spines, elimination of spines, and modifications to the shape of spines [86]. Changes caused by synaptic plasticity may also affect the number of neurotransmitter receptors, thereby altering the strength of signaling to neurotransmitters [87]. Dendritic arborization and spine density form the morphological and structural basis of synaptic plasticity, which is closely correlated to cognitive function [88]. Therefore, the disruption of synaptic plasticity and abnormal spine morphology is often observed in various neurocognitive disorders [89]. Long-term potentiation (LTP), widely considered a biological mechanism of learning and memory, generally refers to the strengthening of synapses [90]. Extensive research proved that long-term depression (LTD) is closely associated with multiple neurodegenerations. Dysregulation of physiological mechanisms between LTD and LTP may lead to cognitive decline, particularly learning and memory deficits. Nevertheless, basal levels of LTD in the hippocampus remain essential for normal cognitive function [91].

*N*-Methyl-D-aspartate receptor (NMDAR) plays a crucial role in synaptic plasticity and cognitive function [92]. For instance, long-term memory is formed by NMDAR-dependent LTP in the hippocampus. As a general rule, NMDAR is present in the presynaptic and postsynaptic sites of dendritic spines, where they are closely associated with regulating glutamate release, LTP, and synaptic plasticity [93, 94]. Extrasynaptic NMDAR is detected on the spine neck, dendritic shaft, or close to the mitochondria. The activation of the extrasynaptic NMDAR causes loss of mitochondrial membrane potential and induces Ca^2+^ neurotoxicity, ultimately resulting in LTD, spinal atrophy, synaptic loss, and apoptosis [93, 95]. Surgical incision–induced pain stimulation appears to have effects on learning and memory functions via NMDA receptor 2B. Painful stimuli induced by surgical trauma decreased the level of cortical NMDA receptor 2B in mice and improved POCD due to painful stimuli by increasing the level of this receptor [42]. This seems to point out that different subtypes of NMDA receptors are performing different roles in cognitive functions.

Increasing evidence suggests that physical exercise improves synaptic plasticity and learning memory impairment in AD models through different pathways [96, 97]. It has been shown that long-term voluntary exercise effectively raises the number of dendritic spines and the level of PSD-95 protein in the hippocampus of mice with AD by inhibiting the abnormal phagocytosis of synapses induced by microglia activation [98]. Interestingly, low-intensity continuous treadmill exercise induced the expression of NMDA receptor 2B in the rat hippocampus, which was suppressed in the high-intensity continuous treadmill exercise group [99]. Resistance exercise prevents the decline of dendritic process complexity and spine density in the hippocampus in POCD animal models, resulting in improved synaptic plasticity and cognitive function in mice [20]. Furthermore, resistance exercise also prevents surgery-induced NMDAR activation [20].In a separate study, appropriate treadmill exercise also reversed the decrease in dendritic branches and spine density in mice undergoing anesthesia and surgery and thus improved post-operative cognitive dysfunction [19].

**Fig. 1:**
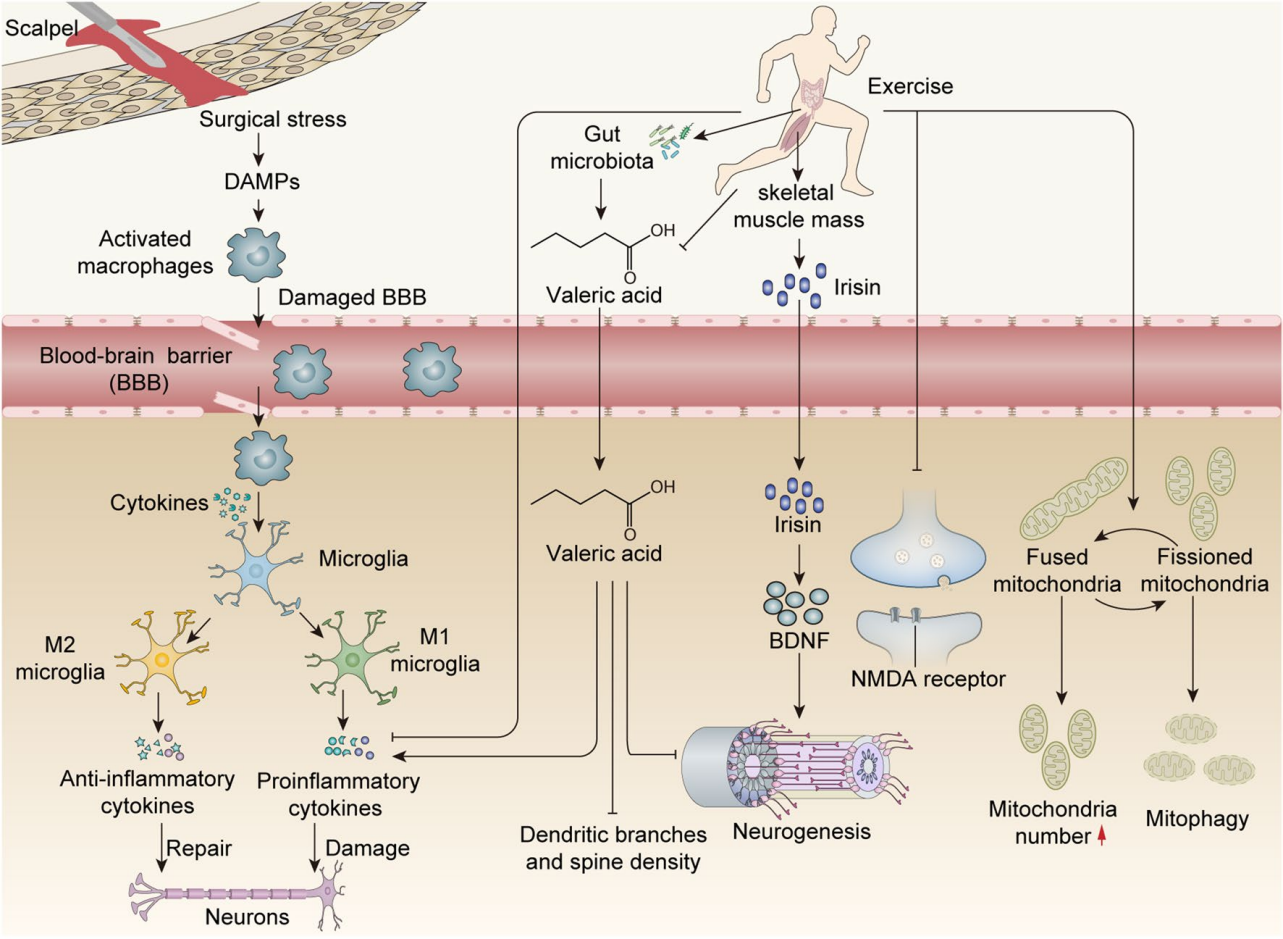
Molecular mechanisms through which exercise may affect cognitive function. Surgical procedures can activate damage-associated molecular patterns (DAMPs), further activating macrophages. Activated macrophages can secrete cytokines through the damaged BBB, further leading to microglia activation in the hippocampus. After being activated, microglia can be divided into M1 and M2 phenotypes. M1-type microglia produce pro-inflammatory cytokines to damage neurons. M2-type microglia secrete anti-inflammatory cytokines participating in tissue repair and extracellular matrix reconstruction. Surgical trauma makes microglia develop M1 polarization, leading to a vicious circle of neuroinflammation progression. Exercise can promote microglia development in M2 polarization and inhibit the level of pro-inflammatory cytokines to promote neuronal recovery. Exercise can inhibit the production of valeric acid by altering the diversity of gut microbiota. Valeric acid produced by gut microbiota inhibited neurogenesis, reduced the dendritic branches and spine density, and increased the production of pro-inflammatory cytokines in the hippocampus. At the same time, exercise can also increase skeletal muscle mass, secrete the muscle factor irisin, and release it into the peripheral blood. Irisin crosses the BBB to promote the production of BDNF and thus induce neurogenesis. Furthermore, exercise regulates mitochondrial dynamics in the hippocampus, thus ensuring mitochondrial quality and reducing mitochondrial abnormalities. Finally, exercise modulates synaptic plasticity by acting on NMDA receptors at the synapse

The benefits of exercise are not only confined to the skeletal muscle but involve the modulation of the gut microbiota, stimulation of neurotrophic factors, maintenance of mitochondrial homeostasis alleviation of neuroinflammation, and improvement in neuroplasticity as well, causing changes in the CNS at the anatomical, cellular, and molecular levels (Fig. 1).

## Appropriate Exercise in the Perioperative Period

Poor physical performance increases mortality and post-operative complications and delays in recovery after surgery [100]. Several clinical studies have shown the beneficial effect of preoperative exercise on post-operative outcomes, including hospital stay, mortality, and complications. Considerable evidence exists which demonstrates improved preoperative physical performance through preoperative exercise, resulting in reduced mortality and complications after major cardiovascular and thoracic surgery [101]. In contrast, Carli et al. reported that patients who completed a more intense exercise program before surgery had worse outcomes [100].

**Fig. 2:**
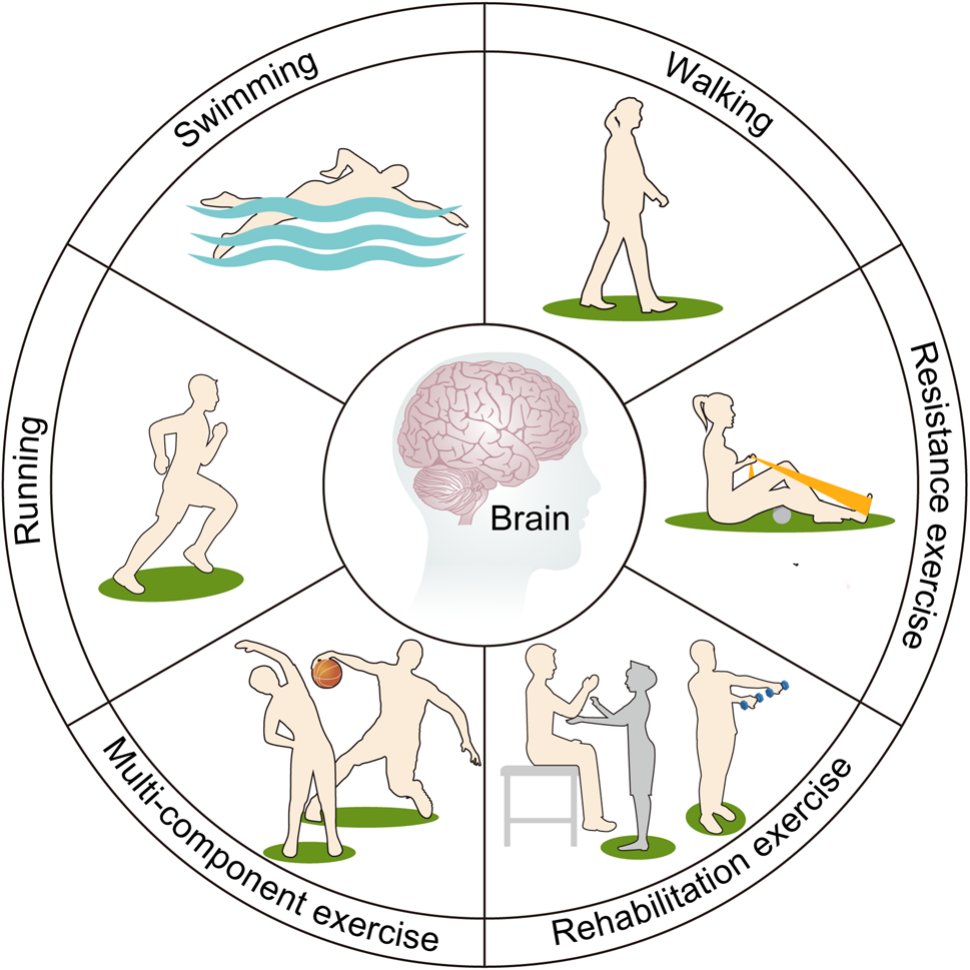
Impact of different types of exercise on the brain. In animal studies, swimming and running have been shown to reduce pro-inflammatory cytokines and regulate gut microbiota, thereby improving post-operative cognitive function. In human studies, walking, multicomponent exercise, and resistance exercise can prevent cognitive decline. Rehabilitation exercise has a protective effect on post-operative cognitive function in elderly patients

Different types of exercise performed by individuals may have different cognitive outcomes. Exercise includes aerobic training and resistance training. Aerobic training can enhance cardiopulmonary function, while resistance exercise can improve skeletal muscle quality. However, both aerobic and resistance training can improve cognition and enhance brain function in older adults [102, 103]. Six months of moderate-intensity aerobic exercise significantly reduced the Alzheimer’s disease Assessment Scale—Cognition (ADAS-Cog) growth score in older adults [104]. In human studies, the effect of aerobic exercise on BDNF is contradictory. Some studies reported that aerobic exercise promoted the expression of BDNF, while others found no changes in BDNF [105]. This appears to be related to the measurement time between BDNF and aerobic exercise, exercise frequency, exercise intensity, age, and gender. Training with adjustable resistance equipment for 12 weeks along with regular exercise improves general learning and memory in older adults with subjective cognitive impairment [106]. The molecular and biological mechanisms of resistance training that help to enhance cognitive function may be different from those of aerobic training. Both aerobic training and resistance exercise improved learning and memory in adult male rats. However, aerobic training preferentially increases BDNF, while resistance training preferentially increases insulin-like growth factor-1 (IGF-1) in the hippocampus [107]. In human studies, hippocampal volume showed a significant increase after aerobic training in older women suffering from probable MCI, whereas resistance exercise failed to do so [108]. Multicomponent exercise might be an effective strategy for preventing cognitive decline and executive dysfunction [109]. In recent literature, a non-linear dose–response relationship exists between overall exercise and cognition, and many types of movement may have clinically important effects at lower doses [110]. A randomized controlled trial has shown that among older adult patients undergoing anesthesia for unilateral total hip replacement, post-operative cognitive therapy combined with rehabilitation exercise has a protective impact on cognitive function compared with the control group [111]. Notably, some types of exercise do not affect cognition, such as cycling and resistance and balance exercise [110]. Although exercise is not suitable for all patients, particularly those with fractures or movement limitations, there are still benefits to be obtained by exercising at the earliest possible time. In summary, both aerobic and resistance exercises have shown great potential in enhancing cognition and improving brain function (Fig. 2).

## Conclusion

This review summarized current clinical evidence of perioperative exercise in improving PNDs and the potential mechanisms by which exercise may influence PNDs. Exercise has been proven to be good for overall health, and each part of the body benefits from different types of exercise, such as aerobic or resistance exercise. Increasing evidence shows that exercise has a beneficial effect on cognitive improvement and brain health [112, 113]. Physical exercise can also reduce anxiety [114] and depression [115]. Moreover, not only does physical exercise reduce the risk of dementia [109], but it also appears to be important in treating the disease [116]. In a separate study, the cognitive decline with age in healthy individuals and those with neurodegenerative diseases was suppressed by regular exercise [117]. Thus, much study-based evidence shows that exercise has a sustained and specific neuroprotective effect.

These studies used animal models to explain the potential benefits of exercise on PNDs. Animal models have contributed to elucidating neuroprotective molecular pathways activated through exercise, suggesting neuroinflammation, synaptic plasticity, and BDNF as key pathways capable of attenuating the phenotypes of neurodegenerative diseases. Nevertheless, only a clearer understanding of the mechanisms by which exercise interventions mediate neuroprotection will allow the design of strategies to prevent PNDs.

Not all levels and type of exercise are beneficial, although exercise can increase the physiological capacity of the heart and skeletal muscle and may improve post-operative complications in older patients as well as the quality of life in patients with PNDs owing to the difference in everyone’s exercise capacity; thus, an individualized exercise program is necessary to maximize the benefits of exercise. In the future, designing reasonable clinical trials, particularly multicenter prospective randomized controlled trials with large samples, is necessary to verify the role of different perioperative exercise in PNDs and its potential benefits on post-operative recovery of older adult patients, which will help to develop more reasonable perioperative exercise strategies.

## Data Availability

Not applicable
